# Imaging in the Evaluation of Endoscopic or Surgical Treatment for Achalasia

**DOI:** 10.1155/2016/2657876

**Published:** 2015-12-27

**Authors:** Diego Palladino, Andrea Mardighian, Marilina D'Amora, Luca Roberto, Francesco Lassandro, Claudia Rossi, Gianluca Gatta, Mariano Scaglione, Guglielmi Giuseppe

**Affiliations:** ^1^Radiology Department, IRCCS “Casa Sollievo della Sofferenza”, San Giovanni Rotondo, Foggia, Italy; ^2^Radiology Department, University of Foggia, Foggia, Italy; ^3^Radiology Department, Second University of Naples, Napoli, Italy; ^4^Radiology Department, “V. Monaldi” Hospital, Napoli, Italy; ^5^Radiology Department, Pineta Grande Medical Center, Castel Volturno, Caserta, Italy

## Abstract

*Purpose.* Aim of the study is to evaluate the efficacy of the endoscopic (pneumatic dilation) versus surgical (Heller myotomy) treatment in patients affected by esophageal achalasia using barium X-ray examination of the digestive tract performed before and after the treatment. *Materials and Methods.* 19 patients (10 males and 9 females) were enrolled in this study; each patient underwent a barium X-ray examination to evaluate the esophageal diameter and the height of the barium column before and after endoscopic or surgical treatment. *Results.* The mean variation of oesophageal diameter before and after treatment is −2.1 mm for surgery and 1.74 mm for pneumatic dilation (OR 0.167, CI 95% 0.02–1.419, and *P*: 0.10). The variations of all variables, with the exception of the oesophageal diameter variation, are strongly related to the treatment performed. *Conclusions.* The barium X-ray study of the digestive tract, performed before and after different treatment approaches, demonstrates that the surgical treatment has to be considered as the treatment of choice of achalasia, reserving endoscopic treatment to patients with high operative risk and refusing surgery.

## 1. Introduction

Achalasia is the most frequent primary motor disorder of the esophagus. It is still a rare disease that may occur in both sexes at any age with a prevalence of less than 1/10,000 and with a new cases' incidence of 0.6–1/100,000 citizens/year [1]. At the base of this disease there is a primitive neuromuscular alteration with a myenteric plexus degeneration causing a pathophysiological disorder consisting in the failure of the lower esophageal sphincter (LES) relaxation during swallowing and the complete loss of peristaltic coordination of the esophagus body [2]. Dysphagia is the typical symptom, consisting in the difficulty in swallowing food; usually the patients have a very long and often unrelated history [3]. Other times the patients may show a sudden onset and, rarely, the symptomatology may be “paradoxical,” more pronounced for liquids than solids. Patients may also feel chest pain and regurgitation. Pain is a less frequent symptom and it is usually observed in the early stages of the disease. It is probably related to the smooth muscles contraction of the esophageal body. Regurgitation is the symptom occurring in later stages, when the esophagus is dilated, and may be misdiagnosed as a gastroesophageal reflux disease, leading to diagnosis delay. In this phase, aspirations of food material may be also present leading to “ab ingestis pneumonia” in 12% of cases [1]. Other times, finally, the only sign of this disease can be a persistent halitosis, due to stagnation of endoesophageal food material. The diagnosis is usually made with X-rays of the digestive tract with barium contrast medium (cm) administration and esophageal manometry [4]. The therapeutic approach may be pharmacological, endoscopic, and surgical [5].

Aim of the study is to evaluate the efficacy of the endoscopic (pneumatic dilation) versus surgical (Heller myotomy) treatment in patients affected by esophageal achalasia through the analysis of parameters deriving from the barium X-ray examination, performed before and after surgical or endoscopic treatment.

## 2. Materials and Methods

The study was approved by the Institutional Ethical Committee and conducted according with the ethical principles of the Declaration of Helsinki. Written informed consent was obtained in all patients.

From January 2009 to December 2014 all the patients referring to our radiology departments for radiological evaluation of achalasia, based on previous esophageal manometry, and planned for surgical or endoscopic treatment were investigated about their clinical history and eligible patients were considered for enrolment in this study. Patients with concomitant systemic neurological and/or rheumatologic disease (e.g., Parkinson disease, scleroderma) were excluded.

Each patient underwent barium X-rays evaluation before and after endoscopic or surgical treatment.

**Figure 1:**
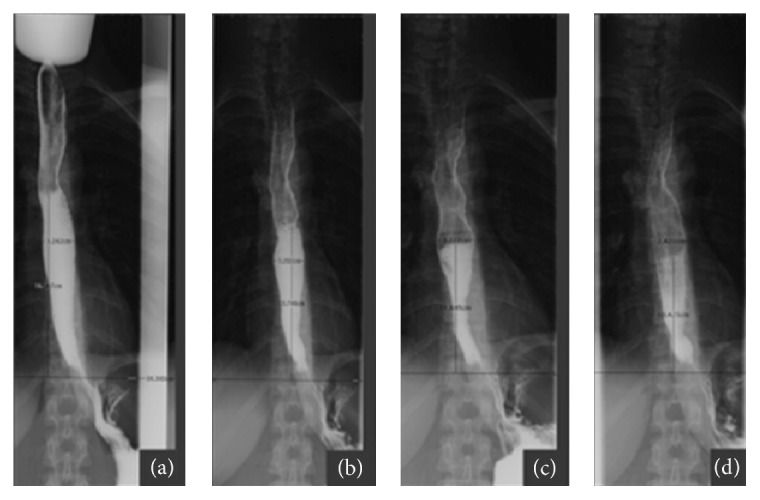
X-rays show the height of barium column 0, 1, 2, and 5 minutes after barium oral administration.

The examination was performed with Siemens AXIOM Luminos DRF equipment. Pronto Bario HD (Bracco, Milan, Italy) has been used as contrast medium; each patient received 98,45 g of powder for oral suspension diluted in 90 mL of water and administered as a single bolus prior to the execution of swellings at 0, 1, 2, and 5 minutes (Figure 1). The esophageal diameter and the height of the barium column were evaluated at the different times after the barium administration. The procedure was successful and was well tolerated in all patients and no complications were reported during the exam execution.

### 2.1. Statistical Analysis

To obtain the logistic model we calculated, for each variable, the mean change was observed after the intervention of the total sample. For each patient were introduced five dichotomous variables (one for each initial variable), assuming value 1 if the reduction found in a particular patient results to be greater than or equal to the average reduction and 0 otherwise. Finally, for each variable, we evaluated the possible relationship with the treatment and we calculated odds ratio.

## 3. Results

Fifty-two patients were initially considered for the study, 12 patients were excluded due to the presence of concomitant systemic neurological disease, 17 did not give their consent, and 4 patients were lost to the evaluation after treatment. Nineteen patients were finally enrolled: 10 males and 9 females, age range was 27–76 y.o. for men and 41–75 for women. Eleven patients underwent surgical Heller myotomy treatment and Dor fundoplication and 8 had endoscopic pneumatic dilation treatment performed, due to the high operative risk and refusal of surgical treatment.

The mean variation of esophageal diameter before and after treatment is −2.1 mm for surgery and 1.74 mm for pneumatic dilation (OR 0.167, CI 95% 0.02–1.419, and *P*: 0.10).  Table 1 shows the variation of esophageal diameter and the height of barium column before and after surgical or endoscopic treatment at 0, 1, 2, and 5 minutes after barium administration.  Table 2 shows the odds ratio calculated with the logistic regression model to demonstrate postoperation mean changes in relation to the two treatments.

## 4. Discussion

The standard in diagnosing and classifying achalasia is represented by the esophageal manometry documenting the impaired relaxation of the LES and the absence or the alteration of peristaltic waves in the distal esophagus [4, 6–9].

Upper endoscopy is usually performed to rule out cancer or a peptic stricture and, particularly in patients older than 50 years with dysphagia and weight loss, attention should be paid on the possible presence of a tumor underlying achalasia (pseudoachalasia) [10, 11]. Cytohistological samples should always be taken in the cardiac region and in the suspicious areas, to find possible neoplastic degeneration [12, 13]. Chest and abdominal CT scan without and with intravenous cm may be helpful in specific, not so common cases [9, 14].

The barium X-ray examination allows to confirm the diagnosis and to assess the degree of esophageal dilation, the axis of the esophagus, and the presence of an associated epiphrenic diverticulum [6], the esophagus appears dilated, aperistaltic, or with uncoordinated peristaltic contractions, sometimes stuffed of food previously ingested and with the “tail mouse” characteristic appearance of the cardiac region [6].

In the early stages, the only sign may be the endoluminal stagnation of cm, with a progressive increase in the height of the barium column until its pressure causes the forced opening and subsequent rapid emptying of the LES.

The cause for an initial reduction of inhibitory neurons in achalasia is unknown; then etiological therapies still do not exist, but only symptomatic treatments [9]. These treatments are designed to solve the lack of LES relaxation. The therapeutic approaches may be pharmacological, endoscopic, or surgical. Drug therapy is not very effective, because, even in the early stages of the treatment, the benefits can be seen only in about 2/3 of the patients, with a chronic drug intake that may cause a reduction in the pharmacological effects with tolerance and addiction phenomena; the possible presence of side effects, such as low blood pressure and related headaches, has to be considered [15]. Some studies have shown a partial efficacy of calcium channel blockers and nitrated derivatives [16], but the use of these medical therapies should be reserved for those patients who cannot tolerate surgical approaches or to who refuse to use them.

Another type of treatment consists of endoscopic therapy that includes the botulinum toxin injection (BTI) and the pneumatic dilation (PD) [17, 18].

BTI is based on a botulinum toxin endoscopic injection in the cardia leading to an inhibition of release of acetylcholine from the myenteric plexus resulting in reduction of smooth muscle contraction of the cardiac region. The effects of a single treatment can persist for six months or more (up to 2-3 years).

The PD consists in the endoscopic introduction, through the mouth, of special dilators, on a metal guide introduced until after the cardia, with the patient maintained under sedation. The dilators consist in cylindrical balloon length of about 12 cm and with variable diameter (2.5 to 4 cm), progressively positioned in the cardiac region. Once placed, it is swollen for 1 minute at 15 PSI pressure. Usually one or two dilations are sufficient to obtain a good result. In 3% of cases, however, there is a cumulative risk of incurring postoperative complications such as tearing and/or perforation of the esophagus. With this method 60–70% of good results may be obtained [19]. The surgical Heller extramucosal myotomy represents the surgical treatment of choice [20]. The intervention consists in the longitudinal section of the cardial esophageal smooth musculature for 6-7 cm; then, an antireflux Dor fundoplication is associated, protecting the esophageal mucosa from the gastroesophageal reflux. According to the available literature, good or excellent results may be obtained in up to 90% of the cases [21].

In our study, the barium X-ray examination of the esophagus (Figure 1) in patients with achalasia, performed before and after the surgical and endoscopic pneumatic treatments, has shown that the average reduction of barium column height observed in patients surgically treated was more noticeable if compared with those treated with pneumatic endoscopic dilation as well as the reduction in the esophageal caliber. The barium X-ray examination was a good test to evaluate the outcome after surgery or endoscopic dilation, being well tolerated and poorly invasive and allowing objectively defining, with a quantitative analysis of the esophageal caliber and morphology. In conclusion, the surgical treatment represents the treatment of choice of achalasia, giving better and more stable results in comparison with endoscopic pneumatic dilation reserved for patients with high operative risk and who refuse surgery; the esophagus X-ray barium study is the modality of choice in the preoperative and postoperative imaging evaluation of these patients.

## Figures and Tables

**Table 1:** Table shows the variation of esophageal diameter and the height of barium column before and after surgical or endoscopic treatment at 0, 1, 2, and 5 minutes after barium administration.

	Surgery			Endoscopy		
	Myotomy			Pneumatic dilation		
	11 patients			8 patients		
	Before	After	Δ	Before	After	Δ
Esophagus diameter (cm)	5.20	3.10	–2.10	4.80	6.54	+1.74
Column baryta height						
0′	23.95	11.9	–12.05	26.50	14.66	–11.89
1′	21.30	7.31	–14.00	25.85	12.61	–13.24
2′	19.64	4.84	–14.79	24.42	11.56	–12.86
5′	16.69	3.75	–12.94	23.06	8.61	–14.45

**Table 2:** Table shows OR, CI 95%, and *P* value calculated with logistic regression model to evaluate statistical significance of the variation between pneumatic dilation and myotomy treatment in patients with diagnosis of achalasia.

	Odd ratio	Confidence interval (95%)		*P* value
*Reduction > mean*				
Esophagus	0.167	0.02	1.419	0.1011
Column baryta height				
0′	0.625	0.093	4.222	0.6297
1′	0.625	0.093	4.222	0.6297
2′	0.429	0.062	2.972	0.3911
5′	0.9	0.133	6.080	0.9139
